# Impact of perfluoroalkyl substances (PFAS) and PFAS mixtures on lipid metabolism in differentiated HepaRG cells as a model for human hepatocytes

**DOI:** 10.1007/s00204-023-03649-3

**Published:** 2023-12-20

**Authors:** Faezeh Sadrabadi, Jimmy Alarcan, Heike Sprenger, Albert Braeuning, Thorsten Buhrke

**Affiliations:** Department of Food Safety, German Federal Institute for Risk Assessment, Max-Dohrn-Str. 8-10, 10589 Berlin, Germany

**Keywords:** PFAS, Mixture effects, Toxicity, Lipid metabolism, HepaRG

## Abstract

Per- and polyfluoroalkyl substances (PFAS) are environmental contaminants with various adverse health effects in humans including disruption of lipid metabolism. Aim of the present study was to elucidate the molecular mechanisms of PFAS-mediated effects on lipid metabolism in human cells. Here, we examined the impact of a number of PFAS (PFOS, PFOA, PFNA, PFDA, PFHxA, PFBA, PFHxS, PFBS, HFPO-DA, and PMPP) and of some exposure-relevant PFAS mixtures being composed of PFOS, PFOA, PFNA and PFHxS on lipid metabolism in human HepaRG cells, an in vitro model for human hepatocytes. At near cytotoxic concentrations, the selected PFAS and PFAS mixtures induced triglyceride accumulation in HepaRG cells and consistently affected the expression of marker genes for steatosis, as well as PPARα target genes and genes related to lipid and cholesterol metabolism, pointing to common molecular mechanisms of PFAS in disrupting cellular lipid and cholesterol homeostasis. PPARα activation was examined by a transactivation assay in HEK293T cells, and synergistic effects were observed for the selected PFAS mixtures at sum concentrations higher than 25 µM, whereas additivity was observed at sum concentrations lower than 25 µM. Of note, any effect observed in the in vitro assays occurred at PFAS concentrations that were at least four to five magnitudes above real-life internal exposure levels of the general population.

**Supplementary Information:**

The online version contains supplementary material available at 10.1007/s00204-023-03649-3.

## Introduction

Per- and polyfluoroalkyl substances (PFAS) are industrial chemicals that have been used since the 1950s for the fabrication of numerous consumer products with surface coatings to provide water- and dirt-repellent properties (Gaines 2023). Due to the chemical nature of the carbon fluorine bond, PFAS are extraordinarily stable against thermal and chemical degradation, and they are also resistant against biological degradation. PFAS belong to the group of persistent organic pollutants (POP) as defined by the Stockholm Convention on POPs, thus being environmental contaminants of high concern (Wang et al. 2017). With respect to human health, toxicity data are available for only a few of the more than 10,000 different existing PFAS. Recently, the European Food Safety Authority (EFSA) published a Scientific Opinion on the risk for human health for those four PFAS for which sufficient toxicity and exposure data were available to conduct a risk assessment: perfluorooctanoic acid (PFOA), perfluorooctane sulfonic acid (PFOS), perfluorohexane sulfonic acid (PFHxS) and perfluorononanoic acid (PFNA) (EFSA 2020). These four PFAS are toxicologically well characterized. They are easily resorbed into the body after, e.g., oral uptake with diet or drinking water, they are not metabolized, and they are slowly excreted with urine and feces. In addition to immunotoxic and developmental effects, EFSA identified an increase of blood serum cholesterol levels as an adverse outcome of exposure to these PFAS in humans. Increased blood serum levels of the liver enzyme alanine aminotransferase (ALT), which is a marker for liver injury, was identified as another adverse outcome of PFAS exposure (EFSA 2020). Numerous in vivo studies with rodents have revealed the hepatotoxic potential of PFAS (NTP 2019a, b, and references therein). At the molecular level, these effects were predominantly associated with a PFAS-mediated activation of the peroxisome proliferator-activated receptor alpha (PPARα) and a subsequent dysregulation of PPARα-dependent lipid metabolism (Bjork and Wallace 2009; Kersten and Stienstra 2017). Numerous animal studies have consistently shown that many PFAS induce hepatocellular hypertrophy and increase liver weights in rodents, which may indicate accumulation of liver fat (Costello et al. 2022). Due to inconclusive experimental data, the steatotic potential of PFAS in humans, however, is not resolved yet. Another main issue of PFAS is that humans are not exposed to single PFAS but rather to many different PFAS compounds. Epidemiological data revealed a widespread presence of PFAS mixtures in human blood across European countries (EFSA 2020). Therefore, it is important to consider possible mixture effects that may result from PFAS exposure (Ojo et al. 2021). Aim of the present study was to elucidate the molecular mechanisms of the PFAS-mediated effects on lipid metabolism in human cells. We have employed fully differentiated human HepaRG cells as a well-established in vitro model for human hepatocytes to comparatively examine the impact of ten different PFAS on lipid metabolism and triglyceride accumulation in these cells. PPARα activation was examined by a transactivation assay in HEK293T cells. In addition to the individual PFAS, three exposure-relevant PFAS mixtures were included in our analysis.

## Material and methods

### Chemicals

Perfluorooctanoic acid (PFOA) (purity ≥ 95%), perfluorooctanesulfonic acid (PFOS) (purity ≥ 98%), perfluorohexanoic acid (PFHxA) (purity ≥ 97%), perfluorohexanesulfonic acid (PFHxS) (purity ≥ 98%), perfluorobutanoic acid (PFBA) (purity ≥ 99%), perfluorobutanesulfonic acid (PFBS) (purity ≥ 97%), perfluorononanoic acid (PFNA) (purity ≥ 97%) and perfluorodecanoic acid (PFDA) (purity ≥ 98%) were obtained from Sigma-Aldrich (Taufkirchen, Germany). Ammonium perfluoro-(2-methyl-3-oxahexanoate) (HFPO-DA, also known as GenX) (purity 99%) was obtained from Apollo Scientific (Cheshire, UK). 3H-perfluoro-3-[(3-methoxypropoxy) propanoic acid] (PMPP, also known as Adona) (purity > 98%) was obtained from Campro Scientific (Berlin, Germany).

Cyclosporine A (purity of 99%) was obtained from Biomol (Hamburg, Germany). Nefazodone (purity ≥ 98%), sodium deoxycholate (purity ≥ 97%), glycocholic acid hydrate (purity ≥ 97%), sodium glycochenodeoxycholate (purity ≥ 97%), sodium chenodeoxycholate (purity ≥ 97%), and sodium glycodeoxycholate (purity ≥ 97%) were purchased from Sigma-Aldrich (Taufkirchen, Germany). Dimethyl sulfoxide (DMSO), GW7647, SR12813 and CITCO were purchased from Merck (Darmstadt, Germany). Rosiglitazone (purity ≥ 98%) was obtained from Cayman (Ann Arbor, USA).

### Cell culture

HepaRG cells were purchased from Biopredic International (Saint Gregoire, France). Cultivation of HepaRG cells is described in detail in Lichtenstein et al. (2020a). Briefly, cells were grown in William’s Medium E with 2 mM glutamine (PAN-Biotech, Aidenbach, Germany), 10% (v/v) fetal bovine serum (FBS) (PAN-Biotech, Aidenbach, Germany), 100 U/ml penicillin and 100 μg/ml streptomycin (Capricorn Scientific, Ebsdorfergrund, Germany), and 5 × 10^−5^ M hydrocortisone hemisuccinate (Sigma-Aldrich, St. Louis, USA) at 37 °C in a humidified atmosphere with 5% CO_2_. The medium was changed every 2 days. After 14 days, 1.7% DMSO was added to the medium to induce differentiation of HepaRG cells, and cells were cultivated for another 14 days. After 4 weeks, the differentiation medium was replaced by treatment medium which has the same composition as the differentiation medium, but with only 2% FBS and 0.5% DMSO. After 48 h, cells were exposed to PFAS dissolved in treatment medium.

HEK293T cells were provided by the European Collection of Cell Cultures (ECACC, Salisbury, UK) and were maintained in Dulbecco’s modified Eagle’s medium (DMEM, PAN-Biotech, Aidenbach, Germany) supplemented with 10% fetal bovine serum (PAN-Biotech, Aidenbach, Germany), 100 U/ml penicillin and 100 μg/ml streptomycin (Capricorn Scientific, Ebsdorfergrund, Germany) at 37 °C in a humidified atmosphere with 5% CO_2_. Cells were passaged at 80–90% confluence and seeded in 96-well plates at 20,000 cells/well density.

### Cellular PFAS exposure considerations

#### Exposure to individual PFAS

For in vitro testing, we selected those PFAS with the highest relevance for human exposure: PFOS, PFOA, PFHxS, PFNA, PFDA, PFHxA, PFBA and PFBS. The sum of these eight PFAS account for more than 90% of the mean PFAS blood serum levels (internal exposure) of the European population and account for more than 80% of the total PFAS found in food (external exposure) in Europe (EFSA 2020). In addition, two next generation PFAS (HFPO-DA and PMPP), which are nowadays used as replacements for PFOA by the industry, were included in our analysis.

#### Exposure to PFAS mixtures

**Fig. 1:**
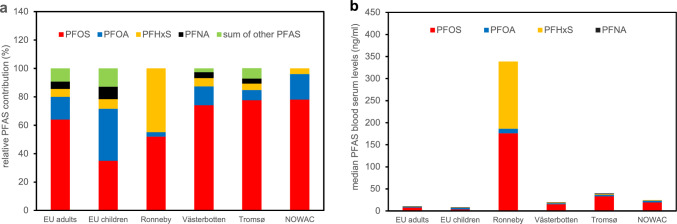
Relative PFAS contribution in human blood samples (**a**) and absolute median blood serum levels (**b**) in the European population (EU adults and EU children, see EFSA 2020) and in a number of cohorts from Sweden and Norway. See text for details and references

To address exposure-relevant PFAS mixtures, we took advantage of the data published by EFSA on the median PFAS blood serum levels of the European population (EFSA 2020). From these internal exposure data, we calculated the relative PFAS contribution in human blood samples, both for adults and children (Fig. 1a). For comparison, the relative PFAS contributions in human blood were also calculated for cohorts from Norway and Sweden. Only PFOS, PFOA and PFHxS were quantified in the blood samples of the Ronneby cohort (*n* = 3418) (Li et al. 2018) and of the NOWAC cohort (*n* = 270) (Rylander et al. 2011). Up to 22 different PFAS were quantified in the blood samples of the Västerbotten cohort (*n* = 187) (Donat-Vargas et al. 2019) and of the Tromsø cohort (*n* = 52) (Nost et al. 2014); however, the dominating PFAS in these samples were PFOS, PFOA, PFHxS and PFNA (Fig. 1a). The relative PFAS contribution in the blood samples of all these cohorts except for the Ronneby cohort was very similar to the distribution in the EU adults (Fig. 1a). When comparing the absolute PFAS blood serum levels, the levels from all cohorts were two- to four-fold higher compared to the EU adults, but the Ronneby cohort is indeed outstanding as the absolute mean PFAS level is more than 30-fold higher than the level of the EU adults (Fig. 1b). Thus, we decided to consider PFAS mixtures according to the distribution (i) in the EU adults which is dominated by PFOS, (ii) in the EU children with an equal contribution of PFOS and PFOA, and (iii) in the Ronneby cohort with an equal contribution of PFOS and PFHxS. Moreover, PFOS, PFOA, PFHxS and PFNA were included into these mixtures as all other PFAS only had a minor contribution to the internal exposure in these cohorts. The composition of the PFAS mixtures examined in the present study is given in Table 1.

**Table 1:** Composition of PFAS mixtures used in the present study

Name of PFAS mixture	Molar ratio of PFOS:PFOA:PFHxS:PFNA
EU adults	0.7:0.18:0.06:0.06
EU children	0.4:0.42:0.08:0.1
Ronneby	0.52:0.03:0.45:0

The molar PFAS ratio of the different mixtures are based on the relative PFAS contribution in blood samples of the European population (EU adults and EU children; EFSA 2020) and of the Ronneby cohort (Li et al. 2018). See also Fig. 1a

### Cytotoxicity

HepaRG Cells were seeded in 96-well plates at a density of 9000 cells/well. Cell differentiation was carried out over a period of 4 weeks as described in the section “cell culture”. HepaRG cells were exposed for 72 h to up to 5 mM of the respective substance except for PFOS, PFOS, PFNA and PFDA that were tested up to a concentration of only 250 µM as it has been reported in a number of previous publications that these long-chain congeners are cytotoxic already at that concentration (Behr et al. 2018, 2020a; Louisse et al. 2020). Besides, due to solubility issues, PFHxS and PMPP were only tested up to a concentration of 1 mM. Cellular viability was determined using 3-(4,5-dimethylthiazol-2-yl)-2,5-diphenyltetrazolium bromide (MTT) assay as described previously (Scharmach et al. 2012). The assay was carried out with six replicates and was repeated in three individual experiments. Medium was used as negative control, while the medium containing 0.01% Triton-X 100 was used as positive control.

### AdipoRed assay

HepaRG cells were seeded and differentiated in 96-well plates (density of 9000 cells/well) and were incubated for 72 h with different concentrations of PFAS. A palmitate/oleate mixture (500 µM each) was used as a positive control. Following treatment, the cell monolayer was washed with phosphate-buffered saline (PBS). Then, 200 μl solution of 5 μg/ml Hoechst 33,342 (Thermo Fischer Scientific, Waltham, USA) freshly prepared in PBS was added to each well for nuclear staining, and 5 μl/well of AdipoRed solution (ready to use; Lonza, Basel, Switzerland) was added for triglyceride staining. After 10-min incubation at 37 °C, fluorescence was measured at Ex 485 nm/Em 572 nm and Ex 350 nm/Em 461 nm for AdipoRed and Hoechst 33,342, respectively, using an Infinite M200 Pro plate reader (Tecan Group, Männedorf, Switzerland). Relative triglyceride levels were referred to solvent control (treatment medium). Three independent biological experiments with six technical replicates for each concentration were performed.

### Cholestatic index

Cholestatic index assessment was performed as described in Gijbels et al. (2021). Briefly, differentiated HepaRG cells were incubated with different concentrations of PFAS with and without a mixture of bile acids for 72 h, with daily renewal of the solutions. The mixture of bile acids consisted in 66 μM glycochenodeoxycholic acid, 20 μM deoxycholic acid, 19.5 μM chenodeoxycholic acid, 19 μM glycodeoxycholic acid, and 17.5 μM glycocholic acid. Nefazodone (30 µM) and cyclosporine A (20 µM) were used as positive controls. After 72 h, cell viability was determined by using the MTT assay (see above). The cholestatic index (CIx) is defined as the ratio of the viability of cells exposed to the test compound and the bile acid mix divided by the viability of cells exposed to the test compound alone (Chatterjee et al. 2014; Gijbels et al. 2021; Hendriks et al. 2016). CIx values were calculated for the different PFAS and for the different test concentrations:

$${\text{CIx}} = \frac{{{\text{viability }}(\%) {\text{ PFAS plus bile acid mix}}}}{{{\text{viability }}(\%) {\text{ PFAS}}}}.$$

Compounds with a CIx value below or equal to 0.8 were considered to have cholestatic potential (Hendriks et al. 2016).

### Nuclear receptor transactivation assays

The luciferase-based nuclear receptor transactivation assays were conducted as described previously (Behr et al. 2020b). HEK293T cells were seeded in 96-well plates at a density of 20,000 cells/well. Cells were transfected with an expression plasmid, pGAL4-hPPARα-LBD, pGAL4-hPXR-LBD or pGAL4-hCAR-LBD, containing a GAL4-dependent DNA-binding domain and a ligand-binding domain of the nuclear receptor hPPARα, hPXR, or hCAR, respectively, and co-transfected with pGAL4-(UAS)_5_-TK-Luc and pcDNA3-Rluc using TransIT-LT1 as transfection reagent (Mirus Bio, Madison, USA). After 4–6 h, cells were exposed to eight different PFAS concentrations ranging from 1 to 250 µM for 24 h. GW7647 (1 µM), SR12813 (10 µM), and CITCO (10 µM) were used as positive controls for the activation of PPARα, PXR, and CAR, respectively. Firefly and *Renilla* luciferase activities were measured via bioluminescence as described by Hampf and Gossen (2006) using an Infinite M200 Pro plate reader (Tecan Group, Männedorf, Switzerland). The firefly luciferase values were normalized to the *Renilla* luciferase values. Three independent biological experiments with three technical replicates for each concentration were performed.

### Gene selection

For gene expression analysis, genes were selected according to published data on gene expression in HepaRG cells. A set of ten marker genes for steatosis was taken from Lichtenstein et al. (2020b). A set of 13 genes associated with lipid and cholesterol metabolism in HepaRG cells including several PPARα and PPARγ target genes was selected according to the publications from Louisse et al. (2020), Pant et al. (2019) and Rogue et al. (2011). Detailed information on the selected genes as well as the primer sequences used for gene expression analysis are given in the Supplementary Information.

### Gene expression analysis

HepaRG cells were seeded in 6-well plates at a density of 200,000 cells/well. Cell differentiation was carried out over a period of 4 weeks as described above. After incubating cells with three different PFAS concentrations ranging from 10 to 1000 µM for 24 h, cells were washed with ice-cold PBS twice, and cells were lysed by adding RLT buffer (RNeasy Mini Kit, Qiagen GmbH, Hilden, Germany). RNA was extracted using the RNeasy Mini kit (Qiagen GmbH, Hilden, Germany) following the manufacturer’s instructions. Quantification of total RNA was done with a spectrophotometer (NanoDrop 1000; Nanodrop Technologies, Wilmington, USA). cDNA was synthesized using the High Capacity Reverse Transcription Kit (Applied Biosystems, Foster City, USA). Real-time qPCR was conducted by using an ABI7900HT instrument (Thermo Fisher, Waltham, MA, USA) with the Maxima SYBR Green/ROX qPCR Master Mix (Thermo Fisher Scientific, Waltham, USA). The thermal cycling program included an initial denaturation step at 95 °C for 15 min, followed by 40 cycles of denaturation at 95 °C for 15 s and primer binding and elongation for 60 s at 60 °C. The procedure was completed with a final elongation step at 60 °C for 15 min and a dissociation curve analysis. Cycle threshold (Ct) values were determined using SDS 2.4.1 software (Life Technologies, Foster City, CA). *18srRNA*, *GAPDH* and *ACTB* were used as housekeeping genes. The geometric mean was computed to analyze gene expression, and relative gene expression levels were determined using the $2^{{ - \Delta \Delta C_{{\text{T}}} }}$ method (Livak and Schmittgen 2001). Three individual experiments were performed.

### Mathematical models

Mixture effects were evaluated using two different models, i.e., the theoretical additivity (TA) and the concentration addition (CA) (Foucquier and Guedj 2015; Cedergreen et al. 2008). The TA model calculates the mixture effect by simple summation of individual effects from each single compound in the mixture. This model was used as described by Weber et al. (2005):

$${E}_{{\text{Mix}}}=1+\sum_{i=1}^{n}\left({E}_{i}\left({c}_{i}\right)-1\right)$$

where *E*_Mix_* is* the effect of the mixture and *E*_*i*_(*c*_*i*_) is the effect of a compound *i* at the concentration *c*_*i*_.

The CA model was used as described by Backhaus et al. (2004):

$${E}_{{\text{Mix}}}={\left(\sum_{i=1}^{n}\frac{{p}_{i}}{{E}_{i}\left({c}_{i}\right)}\right)}^{-1}$$

where *E*_Mix_ is the effect of the mixture and *E*_*i*_(*c*_*i*_) is the effect of a compound *i* at the concentration *c*_*i*_, and* p*_*i*_ the fraction of compound *i* in the mixture.

The possible deviation from additivity was estimated by calculating the model deviation ratio (MDR):

$${\text{MDR}}=\frac{{\text{predicted }} {E}_{{\text{Mix}}}}{{\text{measured }} {E}_{{\text{Mix}}}}.$$

Thresholds for the calculated MDR values were used according to Belden et al. (2007), i.e., MDR < 0.5 indicates effect greater than additivity (synergism), 0.5 ≤ MDR ≤ 2 indicates additivity, while a MDR > 2 indicates effect smaller than additivity (antagonism).

### Data calculation/dose–response modeling

In order to perform the mathematical evaluation of the tested mixtures, one needs to know the effects of single compounds at their corresponding concentrations within the mixture. The concentration levels that were tested for single PFAS in the different endpoints (e.g., PPARα activation) differ from those tested within the mixtures. To circumvent this obstacle, we performed modeling of the dose response curves obtained in experiments with single PFAS compounds to calculate the effects induced by the concentrations tested in the mixtures. Modeling was done using the log(agonist) vs. response–variable slope (four parameters), i.e., Hill slope model, module included in GraphPad Prism. Curve fitting for each single PFAS can be found in Supplementary Fig. 1.

### Statistics and data visualization

GraphPad Prism v.8 (GraphPad Software, La Jolla, USA) was used for statistical analysis of cell viability, triglyceride accumulation, and nuclear receptor activation by doing one-way ANOVA followed by Dunnett’s test (*p < 0.05; **p < 0.01; ***p < 0.001).

Statistical analyses of gene expression data were performed using R (R Core Team 2020) specifically the “rstatix” package (version 0.7.2; Kassambara 2023). To assess the potential treatment effect, one-way ANOVA was performed with subsequent pairwise t-tests based on log2-transformed relative gene expression data. Heatmaps were generated by the R package “ComplexHeatmap” (version 2.14.0; Gu et al. 2016) with default settings. To derive a steatosis prediction from gene expression data, the coefficients of the LASSO regression model (Lichtenstein et al. 2020b) were multiplied with log2 ratios of ten marker genes.

## Results

### Cytotoxicity

**Fig. 2:**
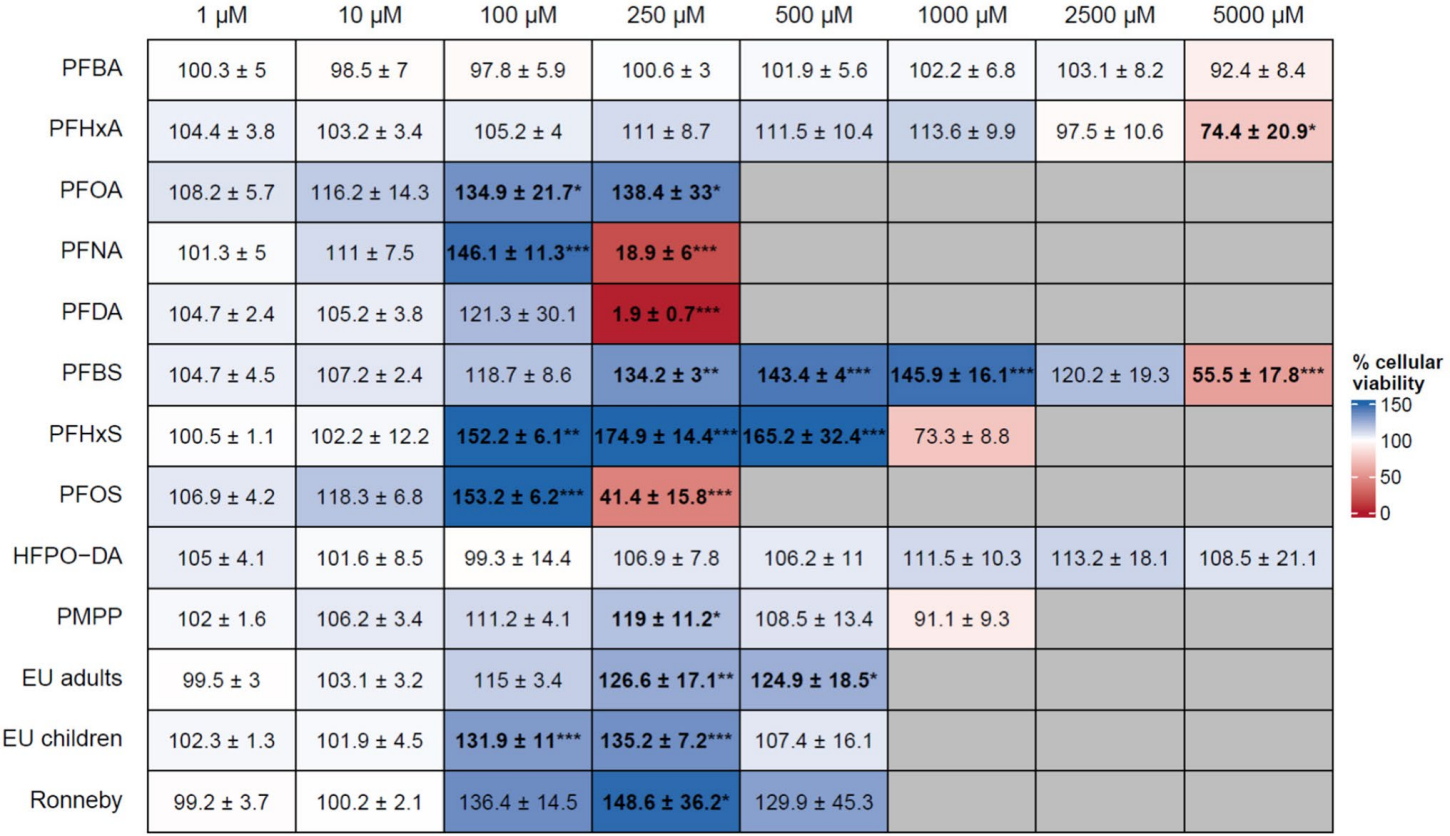
PFAS cytotoxicity. Cellular viability of HepaRG cells after incubation of the cells with different concentrations of different PFAS and PFAS mixtures for 72 h. Cytotoxic PFAS concentrations are highlighted in red. Grey cells indicate that the respective concentration was not tested. The values given are the mean of three independent experiments (mean ± SD) and are normalized to the negative control that was set to 100%. Statistics was done by one-way ANOVA followed by Dunnett’s test (*p < 0.05, **p < 0.01, ***p < 0.001) (color figure online)

According to the MTT assay results, none of the tested PFAS was cytotoxic up to a concentration of 100 µM (Fig. 2). In contrast, with the exception of PFBA, incubation of the cells with the different PFAS and PFAS mixtures resulted in increased MTT values, pointing to a concentration-dependent increase of mitochondrial dehydrogenase activity (Fig. 2). Decreased MTT values were then observed at higher PFAS concentrations at which the respective compound became cytotoxic. Cellular viability was decreased at 250 µM of PFOS, PFNA and PFDA, at 1 mM of PFHxS, and at 5 mM of PFBS and PFHxA. No cytotoxicity was observed for PFOA up to a concentration of 250 µM, for PMPP up to 1 mM, and for PFBA and HFPO-DA up to a concentration of 5 mM (Fig. 2). In summary, (i) PFAS cytotoxicity increased with increasing carbon chain length, (ii) the perfluoroalkyl sulfonic acids (PFSA) displayed a higher cytotoxicity than the respective perfluoroalkyl carboxylic acids (PFCA) congeners with the same carbon chain length, and (iii) the PFOA replacements HFPO-DA and PMPP were less cytotoxic to the cells than PFOA itself. Cytotoxicity of the PFAS mixtures was tested up to a total PFAS concentration in the mixture of 500 µM as PFOS and PFNA, two of the four PFAS in the selected mixtures, were already cytotoxic at a concentration of 250 µM. The three tested mixtures were not cytotoxic to the cells up to a total PFAS concentration of 500 µM.

### Triglyceride accumulation

**Fig. 3:**
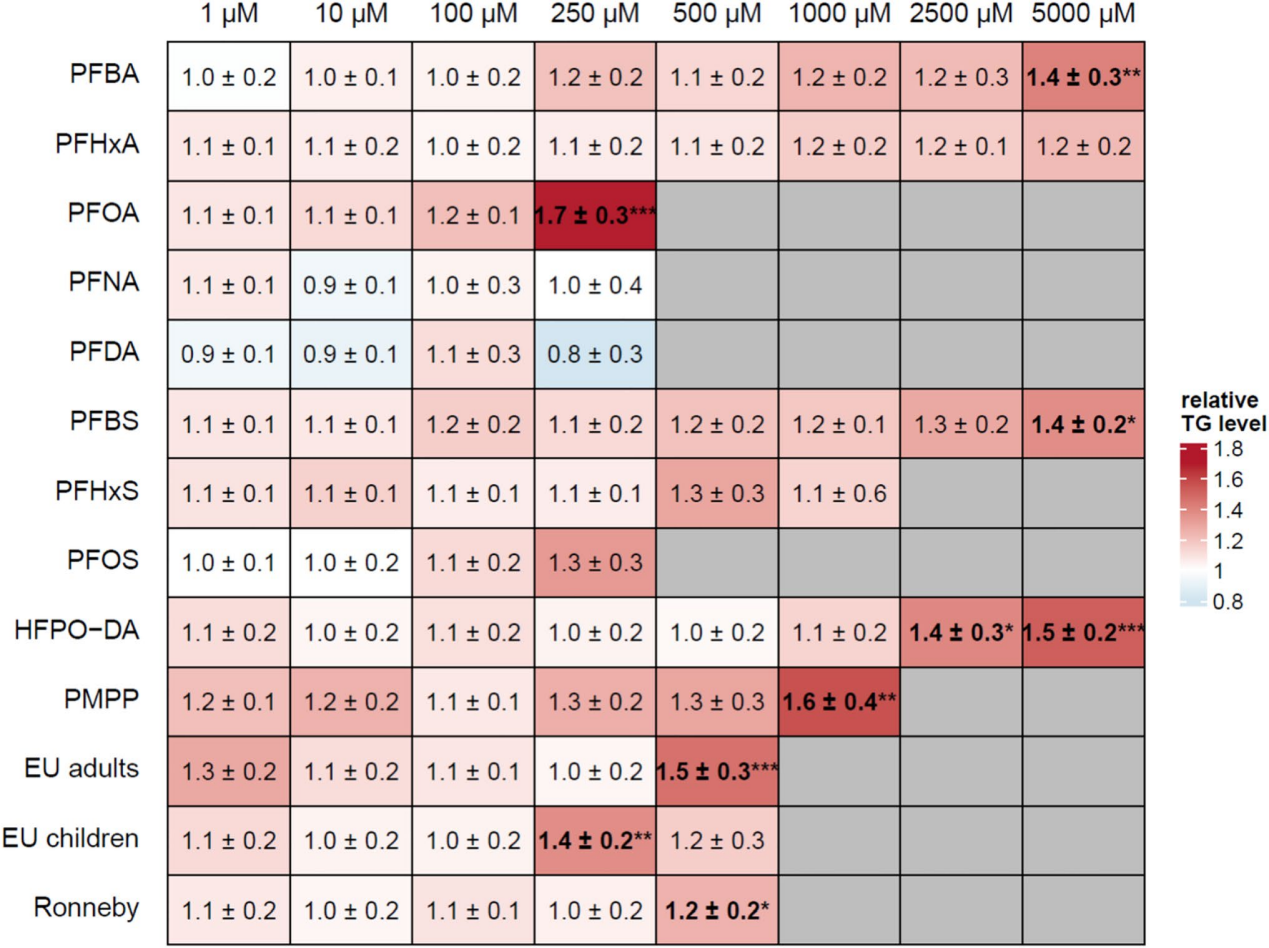
Triglyceride accumulation in HepaRG cells after incubation of the cells with different concentrations of different PFAS and PFAS mixtures for 72 h. In the heatmap, increasing relative triglyceride levels are indicated by increasing intensity of red color. Grey cells indicate that the respective concentration was not tested. The values given are the mean of three independent experiments (mean ± SD) and are normalized to the negative control that was set to 1. Statistics was done by one-way ANOVA followed by Dunnett’s test (*p < 0.05, **p < 0.01, ***p < 0.001) (color figure online)

To examine whether PFAS and PFAS mixtures induce lipid accumulation in HepaRG cells, we took advantage of the AdipoRed assay as a functional assay to determine intracellular triglyceride accumulation. The results of the AdipoRed assay experiments are summarized in Fig. 3. Incubation of HepaRG cells with the positive control palmitate/oleate resulted in a significant 2.5 to 5-fold increase of triglyceride levels compared to the solvent control. In contrast, the individual PFAS and PFAS mixtures only had a minor impact on intracellular triglyceride levels in HepaRG cells. Slight concentration-dependent increases were observed only at concentrations higher than 100 µM that in some cases became statistically significant at the respective highest test concentration. The highest increase was observed for the incubation of the cells with 250 µM PFOA which resulted in a 1.7-fold increase of intracellular triglycerides.

### Steatosis marker gene expression

**Fig. 4:**
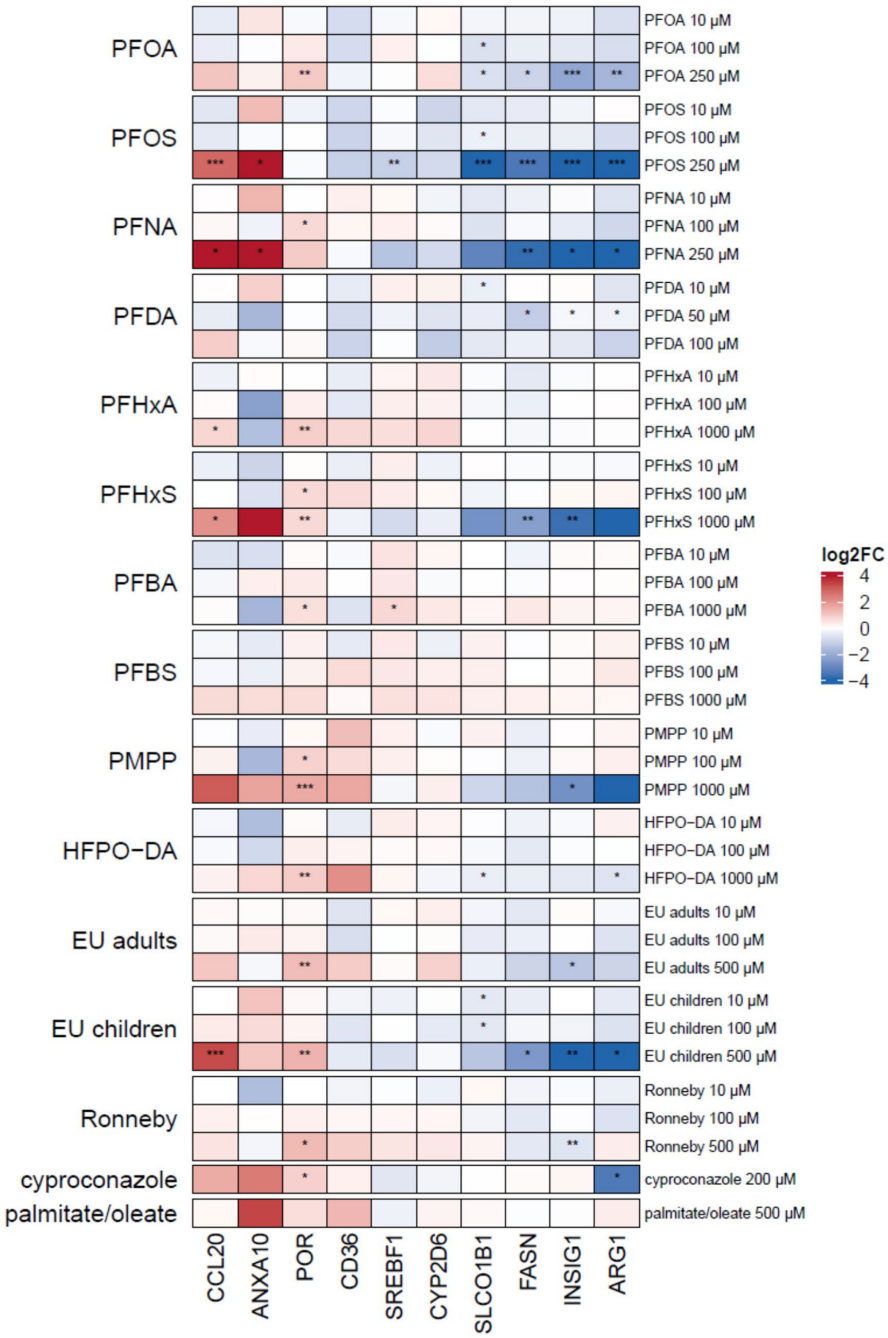
Heatmap of ten steatosis marker gene expression changes in HepaRG cells after incubation of cells with different concentrations of PFAS and PFAS mixtures for 24 h. Median values were calculated from the individual experiments. Fold changes with regard to the respective solvent control were determined and then log2-transformed (log2FC). A positive regulation is indicated by red shades, a negative regulation by blue shades. Significance of gene expression changes is indicated as follows: *p < 0.05, **p < 0.01, ***p < 0.001 (one-way ANOVA followed by Dunnett’s test) (color figure online)

Differentiated HepaRG cells were incubated with the selected PFAS and PFAS mixtures for 24 h, total RNA was extracted from the cells, reverse-transcribed into cDNA and finally subjected to qRT-PCR analysis. In addition to palmitate/oleate, cyproconazole was used as a second positive control as the steatotic potential of this substance has been proven for the HepaRG cell line in a previous study (Lichtenstein et al. 2020b; Luckert et al. 2018). The qRT-PCR results are described in detail in Supplementary Table 2 and are summarized as a heatmap in Fig. 4. At first glance, the expression pattern of the ten marker genes was very similar among the HepaRG incubations with the different PFAS, PFAS mixtures, and the positive controls. The different treatments, e.g., consistently induced gene expression of *ANXA10*, *CCL20* and *POR*, and repressed gene expression of *ARG1*, *FASN* and *INSIG1*. Statistically significant alterations in gene expression were almost exclusively observed for the respective highest, non-cytotoxic test concentration of the different PFAS and PFAS mixtures.

### Steatosis prediction model and correlation with triglyceride accumulation

**Fig. 5:**
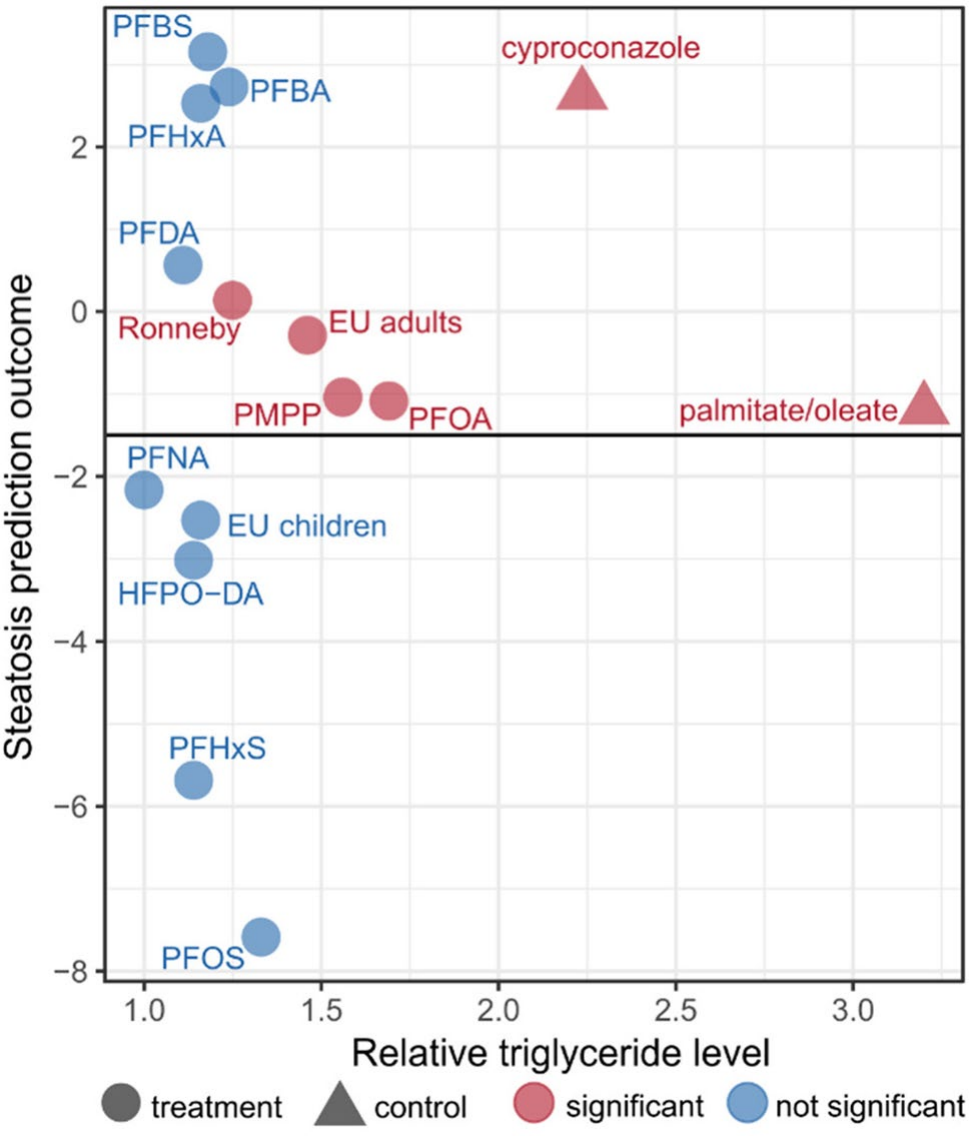
Scatter plot of steatosis prediction outcome vs. relative triglyceride levels. The LASSO regression model was used to calculate a steatosis outcome based on gene expression data. The relative levels on the *x*-axis were determined by the AdipoRed assay for the respective (highest) concentration of each compound also assessed for gene expression. The horizontal line at − 1.5 represents the LASSO-derived artificial boundary line to distinguish between steatosis-positive and negative compounds

The gene expression data were used for a prediction of the steatotic potential of the selected PFAS and PFAS mixtures (Fig. 5). A LASSO regression model was used that yields a numeric value as an outcome for steatosis prediction. According to the model, substances with values higher than − 1.5 are regarded as steatosis-positive (Lichtenstein et al. 2020b). The LASSO regression model that was applied to the log2 ratios of the ten marker genes for the respective highest PFAS concentrations yielded a steatosis-positive prediction (values higher than − 1.5) for eight PFAS/PFAS mixtures (PFOA, PFDA, PFHxA, PFBA, PFBS, PMPP, EU adults and Ronneby) in addition to the two positive controls, and a steatosis-negative prediction (values smaller than − 1.5) for five substances/mixtures (PFOS, PFHxS, PFNA, HFPO-DA, EU children). The results of the steatosis prediction based on the gene expression data were then correlated with the AdipoRed results for triglyceride accumulation (Fig. 5). The scatter plot illustrates that the steatotic potential was—in addition to the two positive controls palmitate/oleate and cyproconazole—correctly predicted for nine PFAS/PFAS mixtures (four positive and five negative). Four false-positive predictions, however, were obtained for PFDA, PFHxA, PFBA and PFBS as these four substances did not induce triglyceride accumulation in HepaRG cells at the respective test concentration.

### Cholestatic potential

**Fig. 6:**
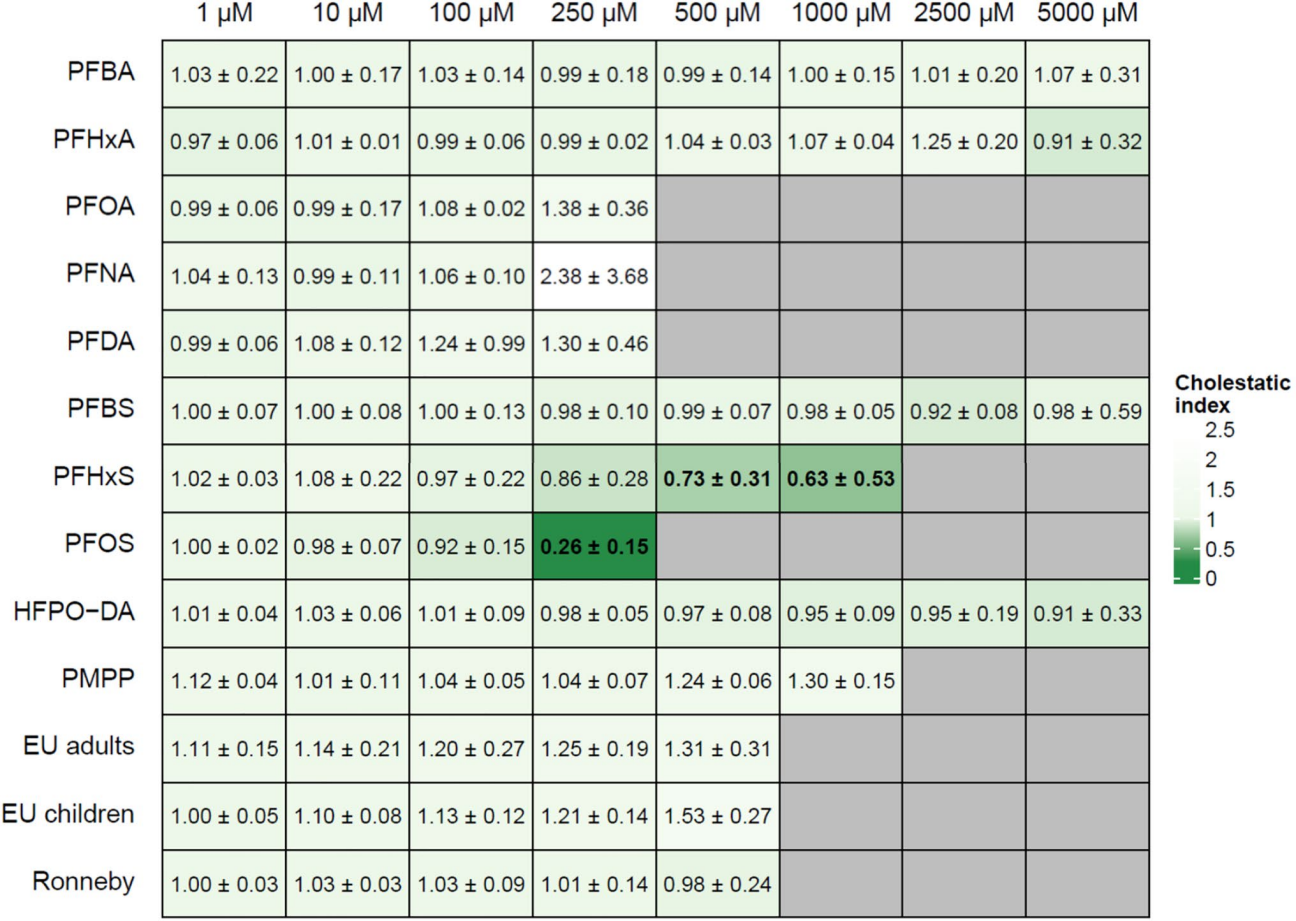
Cholestatic index. HepaRG cells were incubated with different concentrations of different PFAS and PFAS mixtures for 72 h in the presence and in the absence of bile acids. Cellular viability was determined by means of the MTT assay. The cholestatic index is the ratio between the cell viability in the presence of bile acids and the cell viability in the absence of bile acids. The values given are the mean of three independent experiments (mean ± SD). Cholestatic indices smaller than 0.8 are highlighted in bold type and are furthermore indicated by a more intense green color in the heatmap. Grey cells indicate that the respective concentration was not tested (color figure online)

A novel in vitro assay was employed to examine the cholestatic potential of the selected PFAS and PFAS mixtures. HepaRG cells were incubated with the substances and mixtures in the absence as well as in the presence of bile acids, and cholestatic indices were calculated from the results of the MTT assays. The positive controls 20 µM cyclosporine A and 30 µM nefazodone yielded CIx values of 0.68 ± 0.15 and of 0.54 ± 0.09, respectively, indicating their cholestatic potential. In most cases, cell viability as determined for the selected PFAS and PFAS mixtures was independent of the absence or presence of bile acids and thus yielded cholestatic indices of around 1.0 (Fig. 6). CIx values smaller than 0.8 were only obtained for PFOS (at 250 µM) and PFHxS (at 500 µM and 1000 µM), at concentrations which are close to the cytotoxicity level. Thus, in contrast to the positive controls, the cytotoxicity of the selected PFAS and PFAS mixtures is not increased in the presence of bile acids over a broader concentration range, and therefore, these substances are not considered to have a cholestatic potential.

### PPARα activation

**Fig. 7:**
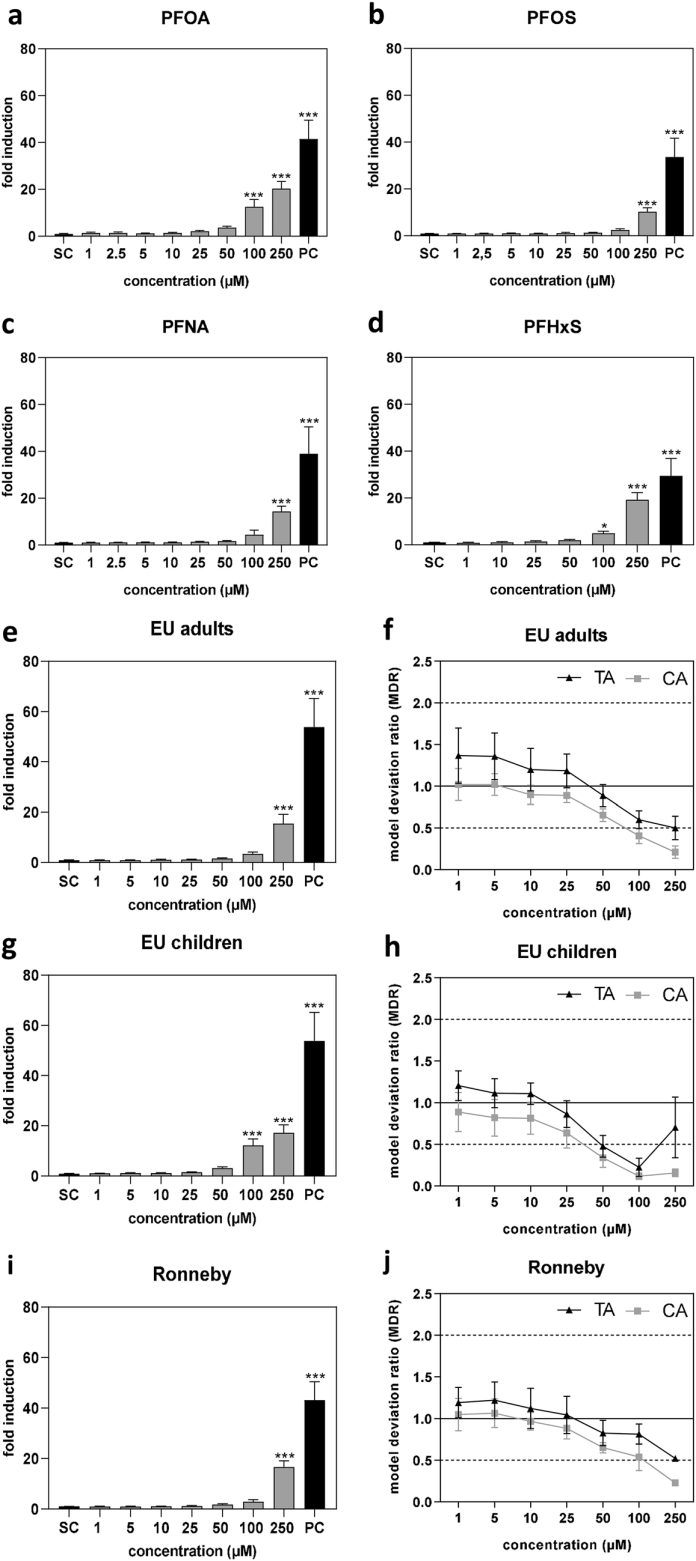
PPARα activation and mixture effects analysis in HEK293T cells after incubation of the cells with different concentrations of PFAS alone or in mixtures for 24 h. Nuclear receptor activation was expressed as the ratio of firefly luciferase signals normalized to *Renilla* luciferase signals. 1 µM GW 7647 was used as positive control (PC) and medium with 1% DMSO was used as solvent control (SC). Activation by single PFAS is depicted in **a** to **d**, whereas mixtures data are shown in **e**, **g**, and **i**. Statistics was done by one-way ANOVA followed by Dunnett’s test (*p < 0.05, and ***p < 0.001). Analysis of mixture effects using the theoretical additivity (TA) method and the concentration addition (CA) concept is depicted in **f**, **h**, and **j**. MDR < 0.5, 0.5 < MDR < 2.0 and MDR > 2.0 indicate respectively synergism, additivity and antagonism. Dashed lines indicate lower and upper limits of additivity. Data represent means ± SD from three independent experiments performed in three technical replicates

PPARα activation by PFAS is the most important molecular initiating event that leads to subsequent key events such as dysregulation of gene expression of PPARα-dependent target genes that may in turn lead to alterations in cholesterol homeostasis, bile acid synthesis and cellular lipid accumulation as adverse outcomes of PFAS at cellular level. We have recently reported that the individual PFAS selected for the present study are capable of activating human PPARα (Behr et al. 2020b). In the present study, we extended the analysis of PFAS-mediated PPARα activation on the PFAS mixtures. The PFAS mixtures are composed of PFOS, PFOA, PFHxS and PFNA, and these four PFAS individually induced PPARα activation at a concentration of 100 µM or above (Fig. 7a–d). A similar PPARα activation pattern was observed for the three PFAS mixtures examined in the present study as these mixtures also activated PPARα at a concentration of 100 µM (sum of the four PFAS) or above (Fig. 7e, g, and i), suggesting effect additivity at first glance. Analysis of putative mixture effects, however, revealed a deviation from additivity for these four PFAS in the selected PFAS mixtures. There was a clear trend of a concentration-dependent decrease of the MDR starting from a PFAS mixture sum concentration of 50 µM (Fig. 7f, h, and j). This accounts for all three mixtures tested in the present study, and for both mathematical models that were employed. Thus, our analysis clearly points to synergistic effects of different PFAS in exposure-relevant PFAS mixtures on PPARα activation, at least at PFAS mixture sum concentrations higher than 25 µM.

### PPARα target gene expression

**Fig. 8:**
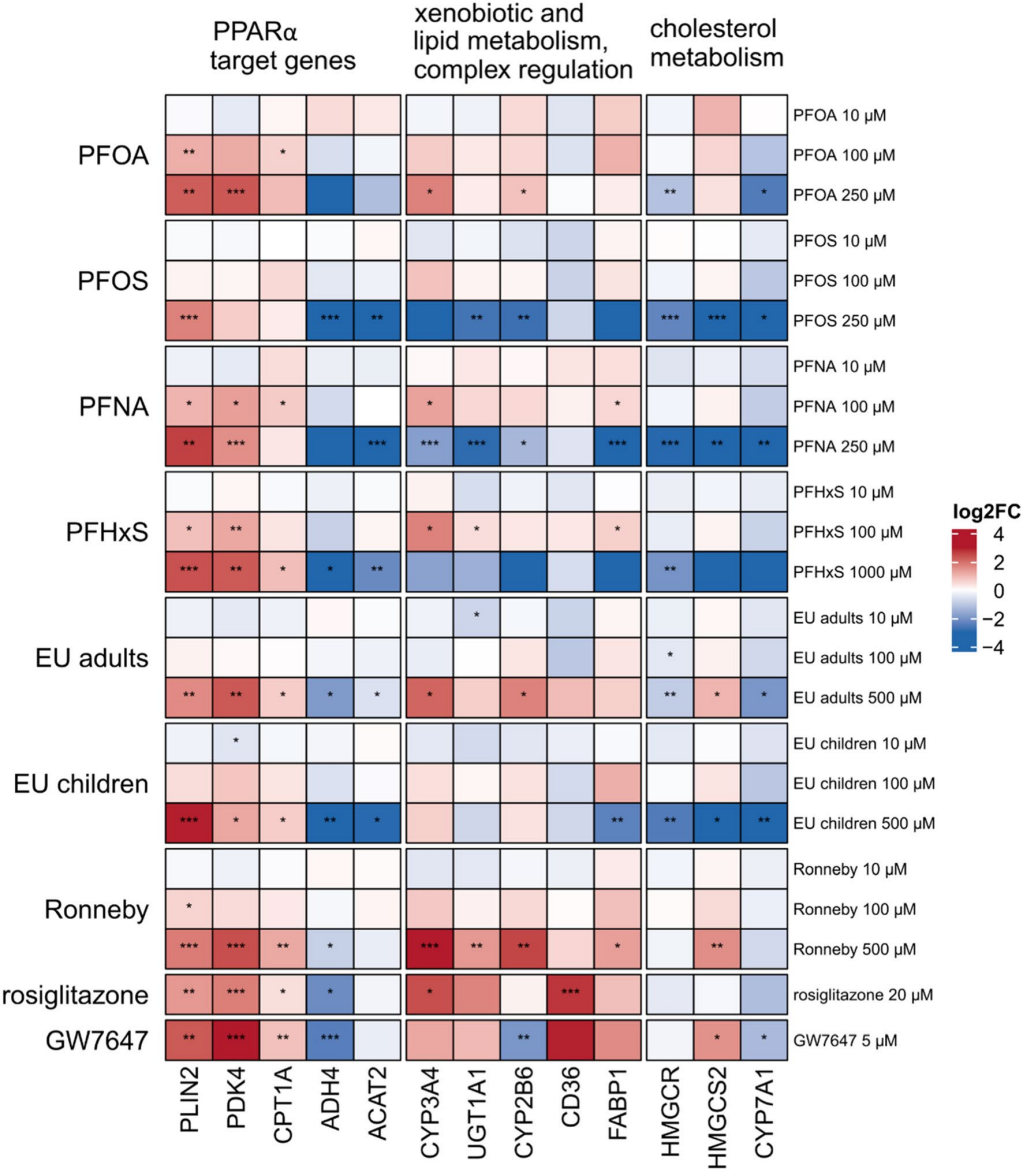
Heatmap of PPARα target gene expression changes in HepaRG cells after incubation of cells with different concentrations of PFAS and PFAS mixtures for 24 h. Median values were calculated from the individual experiments. Fold changes with regard to the respective solvent control were determined and then log2-transformed (log2FC). A positive regulation is indicated by red shades, a negative regulation by blue shades. Significance of gene expression changes is indicated as follows: *p < 0.05, **p < 0.01, ***p < 0.01 (one-way ANOVA followed by Dunnett’s test) (color figure online)

PPARα activation by PFAS and PFAS mixtures was furthermore examined by gene expression analysis. In HepaRG cells, expression of a number of PPARα target genes was consistently upregulated (*PLIN2*, *PDK4*, *CPT1A*) or downregulated (*ADH4*, *ACAT2*) by the synthetic PPARα agonist GW7647, the individual PFAS and the PFAS mixtures (Fig. 8; Supplementary Tables 1 and 3). Gene expression of *CYP2B6*, *CYP3A4* and *UGT1A1* was downregulated by PFOS, PFNA and PFHxS, whereas it was upregulated by PFOA and the mixtures for EU adults and Ronneby, pointing to a more complex regulation of these genes. Notably, gene expression of *CD36* and *FABP1*, the products of which are involved in fatty acid uptake and transport, was upregulated by the PPARα and PPARγ agonists GW7647 and rosiglitazone, respectively, but downregulated by high concentrations of PFAS and PFAS mixtures. Interestingly, genes whose products are involved in cholesterol metabolism (*HMGCR*, *HMGCS2* and *CYP7A1*) were downregulated by PFAS and PFAS mixtures. Overall, the individual PFAS and the PFAS mixtures had a strong and similar effect on the expression of a number of PPARα target genes (*PLIN2*, *PDK4*, *CPT1A1*, *ADH4*, *and ACAT2*) and similarly strongly downregulated *CYP7A1* gene expression.

## Discussion

### Triglyceride accumulation and steatosis potential

Numerous studies have shown that exposure to PFAS is associated with liver damage. Recently, a systematic review identified 85 rodent studies that consistently showed that exposure to either PFOS, PFOA or PFNA results in hepatocellular hypertrophy, increased liver weight, steatosis and liver necrosis in mice and in rats. Moreover, exposure to these three legacy PFAS led to increased serum levels of the liver enzyme ALT, which is a marker for liver injury (Costello et al. 2022). Additional studies reported similar hepatotoxic effects in rodents for further PFAS, e.g., for PFHxS; however, the results of these studies were not always consistent, thus not yet allowing to conclude statistical significant associations. In their systematic review, Costello et al. (2022) also identified 24 epidemiological studies that consistently showed a positive association between blood serum levels of PFOA, PFOS and PFNA and serum ALT levels, indicating that the hepatotoxic effects observed in rodents may also account for humans. The authors concluded that PFAS exposure may contribute to the growing worldwide incidences of non-alcoholic fatty liver disease which has become a public health endemic in the past decades (Mitra et al. 2020). Recently, Sen et al. (2022) examined potential associations between PFAS levels and lipid profiles in samples from human liver biopsies. Interestingly, they found a positive association between levels of PFOA, PFOS and PFNA and triglyceride levels in the liver samples from women, but not from men. In addition to this, they reported correlations between the levels of these three PFAS and the levels of other lipids and of bile acids in the livers of both sexes (Sen et al. 2022). Thus, there is increasing evidence that exposure to PFAS results in alterations in the lipid profiles of human hepatocytes that may finally result in adverse outcomes such as non-alcoholic fatty liver disease. The underlying molecular mechanisms, however, are not resolved yet.

At the molecular level, PFAS-induced PPARα activation is regarded as the most important molecular initiating event that is associated with the observed effects on lipid profiles in hepatocytes. We have shown in an earlier publication that the PFAS examined in the present study activate human PPARα, but no other nuclear receptor that is involved in the regulation of lipid metabolism (Behr et al. 2020b). In a rodent study with *Ppara* knock-out mice it was shown, however, that additional nuclear receptors, e. g. CAR and PPARγ, also contribute to PFAS-mediated effects on gene expression in mouse liver independent of PPARα (Rosen et al. 2017). In a study with mice expressing human PPARα, Schlezinger et al. (2021) showed that PFOA induced liver and serum dyslipidemia when the mice were fed with a cholesterol- and fat-rich diet. PFOA had multiple effects on gene expression and in turn on lipid profiles in the humanized PPARα mice, including increase of total liver triglycerides, supporting the notion that PFOA may have steatotic potential also in humans.

In the present study, we employed the HepaRG cell line to examine the effects of PFAS on human hepatocytes in more detail. Differentiated HepaRG cells display numerous biochemical and morphological features that are very similar to primary human hepatocytes (PHH). Thus, this in vitro system is frequently used, e.g., for toxicity studies. We and others have used HepaRG cells to examine the impact of PFOA and PFOS on lipid metabolism in these cells. In these studies, gene expression analysis has revealed dysregulation of genes associated with lipid metabolism, which is in line with the observed activation of PPARα (Behr et al. 2020b; Louisse et al. 2020, 2023). Similar results have recently been obtained with PHH (Marques et al. 2022). In the present study, we have put a focus on the steatotic potential of PFAS and examined the expression of genes that have recently been shown to be associated with steatosis (Lichtenstein et al. 2020b; Luckert et al. 2018). The steatosis prediction model yielded an inconsistent picture for the PFAS and PFAS mixtures examined in the present study resulting in a steatosis-positive prediction for eight PFAS/PFAS mixtures (PFOA, PFDA, PFHxA, PFBA, PFBS, PMPP, EU adults and Ronneby) and a steatosis-negative prediction for five substances/mixtures (PFOS, PFHxS, PFNA, HFPO-DA, EU children). By correlating the predictions to the AdipoRed results as an experimental readout for triglyceride accumulation, it turned out that the prediction of the steatotic potential was correct for nine PFAS/PFAS mixtures, whereas it also resulted in four seemingly false-positive predictions (Fig. 5). For PFBA and PFBS, however, it has to be noted that the predictions have been conducted with the gene expression data that were obtained with 1000 µM of the respective compound. PFBA and PFBS did not induce triglyceride accumulation at that concentration, but they did at 5000 µM (Fig. 3), indicating that the prediction tool may yield correct predictions at concentrations that are lower than the concentrations required for the adverse outcome of triglyceride accumulation. Thus, alterations in steatosis-related gene expression may be a more sensitive endpoint compared to the functional endpoint (AdipoRed assay). Triglyceride accumulation, on the other hand, may correlate with incubation time in certain cases (Knebel et al. 2019), and the sensitivity of the AdipoRed assay might, therefore, be increased by an elongated incubation time of the cells with the PFAS/PFAS mixtures.

Regarding the functional endpoint, there was a general trend for most PFAS and PFAS mixtures to slightly induce triglyceride accumulation in HepaRG cells at high concentrations, becoming statistically significant in some cases at the respective highest, near cytotoxic test concentration (Fig. 3). Louisse et al. (2020) reported a significant ~ 1.5-fold increase in triglycerides in HepaRG cells when the cells were treated with 100 µM PFOS, 100 µM PFNA or 200 µM PFOA. We observed a significant ~ 1.7-fold increase in triglycerides upon incubation of HepaRG cells with 250 µM PFOA, but no significant increase with 100 µM or 250 µM PFOS. In PHH, legacy and long-chain PFAS did not induce triglyceride accumulation up to a concentration of 25 µM (Marques et al. 2022) which is in line with the results of Louisse et al. (2020) and our own results in HepaRG cells. Interestingly, Marques et al. (2022) reported a ~ 1.5-fold increase in intracellular triglycerides when PHH were incubated with PFBA or PFBS in a concentration range from 0.25 µM up to 25 µM. According to our own data, these short-chain PFAS do not induce triglyceride accumulation in HepaRG cells in that concentration range. However, on the other hand, these two PFAS were predicted to have a steatotic potential (Fig. 5). Taken together, the in vitro results obtained with PHH (Marques et al. 2022) and with HepaRG cells (Louisse et al. 2020; Fig. 3) consistently show that PFAS can induce hepatocellular triglyceride accumulation. The increase to a level of ~ 1.5-fold, however, is moderate compared to strong steatosis inducers such as 500 µM palmitate/oleate and is only observed at high PFAS concentrations (> 100 µM) which are several magnitudes above the mean internal PFAS exposure of the population (< 10 nM). This also accounts for highly exposed populations such as the Ronneby cohort which has a mean internal PFAS exposure of < 1 µM. Thus, it can be assumed that the real-life exposure to PFAS—and even in a longtime high exposure as, e.g., seen for the Ronneby cohort—might not be sufficient to initiate the onset of NAFLD as an adverse outcome in humans. It might, however, be speculated whether the exposure to PFAS could increase the severity of an existing NAFLD.

### Cholesterol homeostasis

In addition to their steatotic potential, many PFAS are known to impact cholesterol homeostasis. Interestingly, several studies with rodents have consistently shown a negative association between PFAS blood serum levels and total serum cholesterol (NTP 2019a, b), whereas epidemiological studies revealed a positive association between PFAS blood serum levels and total serum cholesterol in humans (EFSA 2018; Dong et al. 2019; Jain and Ducatman 2019; Nelson et al. 2010). The PFAS-mediated increase in total serum cholesterol was identified as one important adverse outcome of exposure to PFOS, PFOA, PFNA and PFHxS for humans (EFSA 2020). In the present study, we employed a novel in vitro assay with HepaRG cells that was designed to identify the cholestatic potential of a given test substance. In contrast to the well-known cholestasis inducers cyclosporine A and nefazodone, none of the tested PFAS except for PFOS and PFHxS at high, nearly cytotoxic concentrations yielded a CIx value smaller than 0.8. Thus, according to the results of that in vitro test, these PFAS cannot be labeled “cholestatic”. In an earlier study, however, we have clearly shown an impact of PFOA and PFOS on cholesterol homeostasis and bile acid synthesis in HepaRG cells (Behr et al. 2020a). In that study we have shown that both PFOA and PFOS impair bile acid synthesis by inhibiting expression of *CYP7A1*, the gene encoding the key enzyme catalyzing the rate-limiting step of bile acid synthesis from cholesterol. Moreover, PFOA and PFOS affected the expression of a number of genes involved in cholesterol synthesis and transport (e.g., *SREBP1, ABCG1, ABCG5,* and *ABCG8*) and in bile acid synthesis, transport and detoxification (e.g., *CYP27A1, SLC10A1, BAAT, UGT2B4, UGT2B7,* and *SULT2A1*) in a similar manner as cyclosporine A. These two PFAS, however, had a different effect on HepaRG cell morphology compared to cyclosporine A. Whereas cyclosporine A induced a restriction of bile acid canaliculi, PFOA and PFOS induced an opposite effect, namely a dilation of the bile acid canaliculi (Behr et al. 2020a). This leads to the conclusion that the novel in vitro assay is obviously not sensitive enough to identify weakly cholestatic substances such as PFAS, although they have a clear impact on cholesterol homeostasis and bile acid synthesis. Alternatively, the in vitro assay fails to detect cholestatic substances when the underlying molecular mechanism is different from that of classical cholestatic compounds such as cyclosporine A which inhibits bile acid excretion via the bile salt export pump (BSEP). According to the AOP for cholestasis, inhibition of BSEP has been recognized as the molecular initiating event for the onset of drug-induced cholestasis (Vinken et al. 2013).

### PPARα activation and target gene expression

In the present study, we characterized the effects of three real-life PFAS mixtures in comparison to the individual PFAS. Overall, the effects of the mixtures on cytotoxicity, gene expression and triglyceride accumulation were very similar to those of the single compounds. However, a more detailed analysis on potential mixture effects, which was conducted regarding the results on PPARα activation, showed that at concentrations below 25 µM, the four PFAS that were present in the PFAS mixtures (PFOS, PFOA, PFHxS and PFNA) additively induced activation of human PPARα. However, a deviation from additivity in the way of synergism was observed at concentrations higher than 25 µM. In comparable studies on mixture effects of mainly binary PFAS mixtures, similar results were obtained regarding transactivation of mouse PPARα (Wolf et al. 2014) and of the PPARα homolog cloned from *Gadus morhua* (Atlantic cod) (Soderstrom et al. 2022). In the latter study, the authors showed with double-ligand docking analyses and molecular dynamics calculations that the PPARα ligand-binding domain possesses an allosteric binding site for PFAS in addition to the canonical ligand-binding pocket. In their calculations, they showed that, e.g., binding of PFOS to that allosteric binding site enhances PPARα activation mediated by binding of PFOA to the ligand-binding pocket. This could explain the observed synergistic effects of PFAS mixtures at high concentrations. In contrast to this finding, other groups have not observed synergistic effects of PFAS mixtures in PPARα reporter gene assays. Carr et al. (2013) even observed antagonistic effects using binary mixtures of PFOA, PFNA, PFOS and PFHxS in a PPARα reporter gene assay that was based on mouse PPARα.

In addition to PPARα-dependent reporter gene assays, gene expression of PPARα target genes is commonly used to examine PPARα activation. In the present study, we have selected a set of genes that have previously been shown to be regulated in HepaRG cells by the PPARα agonist GW7647 (Louisse et al. 2020; Pant et al. 2019) or the PPARγ agonist rosiglitazone (Rogue et al. 2011). Our results clearly show that a number of PPARα target genes (*PLIN2*, *PDK4*, *CPT1A*, *ADH4*, and *ACAT2*) are affected by individual PFAS and PFAS mixtures similarly as by GW7647, thereby supporting the notion that PFAS activate PPARα in HepaRG cells. Recently, Louisse et al. (2023) reported a strong impact of PFOS on gene expression in HepaRG cells, in particular on PPARα target genes and on genes related to cholesterol biosynthesis, which is in line with our own data. In that study, the authors combined AdipoRed data on triglyceride accumulation with gene expression data for ten selected genes associated with lipid metabolism to derive relative potency factors for 18 selected PFAS. The authors concluded that *OAT5* expression was the most suitable readout to derive relative potency factors for PFAS in HepaRG cells, and that the HepaRG cell model is a suitable screening tool to study hepatoxic effects in vitro (Louisse et al. 2023).

Regarding PFAS mixture effects, the selected PFAS mixtures affected expression of PPARα target genes and of genes associated with cholesterol metabolism in a similar manner compared to the individual PFAS. There were, however, notable differences regarding expression of genes that underlie a more complex regulation. As an example, the Ronneby mixture strongly induced gene expression of *CYP3A4* and *CYP2B6*, whereas expression of these two genes was repressed by PFOS and PFHxS (Fig. 8). Thus, the Ronneby mixture that is mainly composed of PFOS and PFHxS (Fig. 1) obviously had the opposite effect on *CYP3A4* and *CYP2B6* gene expression than the two individual PFAS. Although it has been shown that expression of these two genes is affected by PPARα and PPARγ agonists (Rogue et al. 2011; Pant et al. 2019) as well as by PFAS (Behr et al. 2020a; Fig. 8), *CYP3A4* and *CYP2B6* gene expression is mainly regulated by PXR and CAR, respectively. In the case of PXR, it has been shown that this nuclear receptor can be activated by mixtures of pharmaceutical and environmental compounds although it is not activated by the individual compounds (Delfosse et al. 2015). In the case of PFAS, however, there was no obvious difference in the activation of PXR and CAR by PFOS, PFHxS, and the Ronneby mixture (Supplementary Fig. 2). Thus, it has to be noted that PFAS mixtures may have opposite effects on gene expression in HepaRG cells compared to the respective individual PFAS. Comprehensive transcriptomic studies will be required to elucidate the underlying molecular mechanisms for these PFAS mixture effects on gene expression.

In view of these observations, it would be desirable to conduct an analysis of potential mixture effects as it was done for the PPARα transactivation data. Due to the limited number of PFAS concentrations that were selected for gene expression analysis, however, our own gene expression data were not suitable to conduct a mathematical modeling to identify additive, synergistic or antagonistic effects. In a large study with human liver spheroids, Addicks et al. (2023) examined several mixtures being composed of PFBA, PFPeA, PFHxA, PFHpA, PFOA, PFNA, PFDA, PFUnA, PFBS, PFHxS, PFOS, PFOSA, FtS 6-2 and FtS 8-2 and concluded that these PFAS have additive effects in mixtures, sometimes—depending on the specific mixture—with a trend to synergism or antagonism. Conley et al. (2023) concluded dose additivity from gene expression signatures in livers from rats that had been treated with different PFAS mixtures. By using the rat hepatoma cell line FaO, Bjork et al. (2021) concluded that binary mixtures of PFOS, PFOA, PFHxS, PFBA and PFBS have antagonistic effects in upregulating the PPARα target gene *Ehhadh*. Thus, taken together, the different studies on PPARα activation and on PPARα target gene expression available so far do not give a consistent picture whether PFAS mixtures have an additive, a synergistic or an antagonistic effect on PPARα activation. The inconsistent results may be due to the different biological models (human vs. rat vs. mouse; primary cells vs. cell line) that were used in the different studies.

## Conclusions

In conclusion, the individual PFAS and PFAS mixtures examined in the present study have an impact on lipid metabolism in HepaRG cells. Incubation of the cells with individual PFAS and PFAS mixtures resulted in triglyceride accumulation, and in a consistent dysregulation of marker genes for steatosis, of PPARα target genes and of additional genes involved in lipid and cholesterol metabolism, e.g., *CYP7A1*. Regarding PPARα activation, synergistic effects were observed for the selected exposure-relevant PFAS mixtures being composed of PFOS, PFOS, PFNA and PFHxS. It has to be noted, however, that any effect described in the present study only occurred at concentrations that were at least four to five magnitudes above real-life internal PFAS exposure levels of the general population and still two orders of magnitudes above internal PFAS exposure levels of highly exposed populations such as in the Ronneby cohort.

### Supplementary Information

Below is the link to the electronic supplementary material.

Supplementary file1 (XLSX 623 kb)

Supplementary file2 (DOCX 131 kb)

Supplementary file3 (DOCX 21 kb)

Supplementary file4 (XLSX 47 kb)

Supplementary file5 (XLSX 32 kb)

## Data Availability

Raw data are available in the supplements or on request.

## References

[CR1] Addicks GC, Rowan-Carroll A, Reardon AJF (2023). Per- and polyfluoroalkyl substances (PFAS) in mixtures show additive effects on transcriptomic points of departure in human liver spheroids. Toxicol Sci.

[CR2] Backhaus T, Faust M, Scholze M, Gramatica P, Vighi M, Grimme LH (2004). Joint algal toxicity of phenylurea herbicides is equally predictable by concentration addition and independent action. Environ Toxicol Chem.

[CR3] Behr AC, Lichtenstein D, Braeuning A, Lampen A, Buhrke T (2018). Perfluoroalkylated substances (PFAS) affect neither estrogen and androgen receptor activity nor steroidogenesis in human cells in vitro. Toxicol Lett.

[CR4] Behr AC, Kwiatkowski A, Stahlman M (2020). Impairment of bile acid metabolism by perfluorooctanoic acid (PFOA) and perfluorooctanesulfonic acid (PFOS) in human HepaRG hepatoma cells. Arch Toxicol.

[CR5] Behr AC, Plinsch C, Braeuning A, Buhrke T (2020). Activation of human nuclear receptors by perfluoroalkylated substances (PFAS). Toxicol In Vitro.

[CR6] Belden JB, Gilliom RJ, Lydy MJ (2007). How well can we predict the toxicity of pesticide mixtures to aquatic life?. Integr Environ Assess Manag.

[CR7] Bjork JA, Wallace KB (2009). Structure-activity relationships and human relevance for perfluoroalkyl acid-induced transcriptional activation of peroxisome proliferation in liver cell cultures. Toxicol Sci.

[CR8] Bjork JA, Dawson DA, Krogstad JO, Wallace KB (2021). Transcriptional effects of binary combinations of PFAS in FaO cells. Toxicology.

[CR9] Carr CK, Watkins AM, Wolf CJ, Abbott BD, Lau C, Gennings C (2013). Testing for departures from additivity in mixtures of perfluoroalkyl acids (PFAAs). Toxicology.

[CR10] Cedergreen N, Christensen AM, Kamper A (2008). A review of independent action compared to concentration addition as reference models for mixtures of compounds with different molecular target sites. Environ Toxicol Chem.

[CR11] Chatterjee S, Richert L, Augustijns P, Annaert P (2014). Hepatocyte-based in vitro model for assessment of drug-induced cholestasis. Toxicol Appl Pharmacol.

[CR12] Conley JM, Lambright CS, Evans N (2023). Dose additive maternal and offspring effects of oral maternal exposure to a mixture of three PFAS (HFPO-DA, NBP2, PFOS) during pregnancy in the Sprague-Dawley rat. Sci Total Environ.

[CR13] Costello E, Rock S, Stratakis N (2022). Exposure to per- and polyfluoroalkyl substances and markers of liver injury: a systematic review and meta-analysis. Environ Health Perspect.

[CR14] Delfosse V, Dendele B, Huet T (2015). Synergistic activation of human pregnane X receptor by binary cocktails of pharmaceutical and environmental compounds. Nat Commun.

[CR15] Donat-Vargas C, Bergdahl IA, Tornevi A (2019). Associations between repeated measure of plasma perfluoroalkyl substances and cardiometabolic risk factors. Environ Int.

[CR16] Dong Z, Wang H, Yu YY, Li YB, Naidu R, Liu Y (2019). Using 2003–2014 U.S. NHANES data to determine the associations between per- and polyfluoroalkyl substances and cholesterol: trend and implications. Ecotoxicol Environ Saf.

[CR17] EFSA (2018) EFSA CONTAM Panel (EFSA Panel on Contaminants in the Food Chain), Knutsen HK, Alexander J, Barreg_ard L, Bignami M, Br€uschweiler B, Ceccatelli S, Cottrill B, Dinovi M, Edler L, Grasl-Kraupp B, Hogstrand C, Hoogenboom LR, Nebbia CS, Oswald IP, Petersen A, Rose M, Roudot A-C, Vleminckx C, Vollmer G, Wallace H, Bodin L, Cravedi J-P, Halldorsson TI, Haug LS, Johansson N, van Loveren H, Gergelova P, Mackay K, Levorato S, van Manen M, Schwerdtle T. Scientific Opinion on the risk to human health related to the presence of perfluorooctane sulfonic acid and perfluorooctanoic acid in food. EFSA J 16(12):5194 (2018). 10.2903/j.efsa.2018.5194 10.2903/j.efsa.2018.5194 PMC7009575 32625773

[CR18] EFSA (2020) EFSA CONTAM Panel (EFSA Panel on Contaminants in the Food Chain), Schrenk D, Bignami M, Bodin L, Chipman JK, del Mazo J, Grasl-Kraupp B, Hogstrand C, Hoogenboom LR, Leblanc J-C, Nebbia CS, Nielsen E, Ntzani E, Petersen A, Sand S, Vleminckx C, Wallace H, Barreg_ard L, Ceccatelli S, Cravedi J-P, Halldorsson TI, Haug LS, Johansson N, Knutsen HK, Rose M, Roudot A-C, Van Loveren H, Vollmer G, Mackay K, Riolo F, Schwerdtle T. Scientific opinion on the risk to human health related to the presence of perfluoroalkyl substances in food. EFSA J 18(9):6223 (2020). 10.2903/j.efsa.2020.6223 10.2903/j.efsa.2020.6223 PMC7507523 32994824

[CR19] Foucquier J, Guedj M (2015). Analysis of drug combinations: current methodological landscape. Pharmacol Res Perspect.

[CR20] Gaines LGT (2023). Historical and current usage of per- and polyfluoroalkyl substances (PFAS): a literature review. Am J Ind Med.

[CR21] Gijbels E, Devisscher L, Vinken M (2021). Testing in vitro tools for the prediction of cholestatic liver injury induced by non-pharmaceutical chemicals. Food Chem Toxicol.

[CR22] Gu Z, Eils R, Schlesner M (2016). Complex heatmaps reveal patterns and correlations in multidimensional genomic data. Bioinformatics.

[CR23] Hampf M, Gossen M (2006). A protocol for combined Photinus and Renilla luciferase quantification compatible with protein assays. Anal Biochem.

[CR24] Hendriks DF, Fredriksson Puigvert L, Messner S, Mortiz W, Ingelman-Sundberg M (2016). Hepatic 3D spheroid models for the detection and study of compounds with cholestatic liability. Sci Rep.

[CR25] Jain RB, Ducatman A (2019). Roles of gender and obesity in defining correlations between perfluoroalkyl substances and lipid/lipoproteins. Sci Total Environ.

[CR26] Kassambara A (2023) rstatix: Pipe-friendly framework for basic statistical tests, 0.7.2

[CR27] Kersten S, Stienstra R (2017). The role and regulation of the peroxisome proliferator activated receptor alpha in human liver. Biochimie.

[CR28] Knebel C, Buhrke T, Sussmuth R, Lampen A, Marx-Stoelting P, Braeuning A (2019). Pregnane X receptor mediates steatotic effects of propiconazole and tebuconazole in human liver cell lines. Arch Toxicol.

[CR29] Li Y, Fletcher T, Mucs D (2018). Half-lives of PFOS, PFHxS and PFOA after end of exposure to contaminated drinking water. Occup Environ Med.

[CR30] Lichtenstein D, Luckert C, Alarcan J (2020). An adverse outcome pathway-based approach to assess steatotic mixture effects of hepatotoxic pesticides in vitro. Food Chem Toxicol.

[CR31] Lichtenstein D, Mentz A, Schmidt FF (2020). Transcript and protein marker patterns for the identification of steatotic compounds in human HepaRG cells. Food Chem Toxicol.

[CR32] Livak KJ, Schmittgen TD (2001). Analysis of relative gene expression data using real-time quantitative PCR and the 2(-Delta Delta C(T)) method. Methods.

[CR33] Louisse J, Rijkers D, Stoopen G (2020). Perfluorooctanoic acid (PFOA), perfluorooctane sulfonic acid (PFOS), and perfluorononanoic acid (PFNA) increase triglyceride levels and decrease cholesterogenic gene expression in human HepaRG liver cells. Arch Toxicol.

[CR34] Louisse J, Fragki S, Rijkers D (2023). Determination of in vitro hepatotoxic potencies of a series of perfluoroalkyl substances (PFASs) based on gene expression changes in HepaRG liver cells. Arch Toxicol.

[CR35] Luckert C, Braeuning A, de Sousa G (2018). Adverse outcome pathway-driven analysis of liver steatosis in vitro: a case study with cyproconazole. Chem Res Toxicol.

[CR36] Marques E, Pfohl M, Wei W (2022). Replacement per- and polyfluoroalkyl substances (PFAS) are potent modulators of lipogenic and drug metabolizing gene expression signatures in primary human hepatocytes. Toxicol Appl Pharmacol.

[CR37] Mitra S, De A, Chowdhury A (2020). Epidemiology of non-alcoholic and alcoholic fatty liver diseases. Transl Gastroenterol Hepatol.

[CR38] Nelson JW, Hatch EE, Webster TF (2010). Exposure to polyfluoroalkyl chemicals and cholesterol, body weight, and insulin resistance in the general U.S. population. Environ Health Perspect.

[CR39] Nost TH, Vestergren R, Berg V, Nieboer E, Odland JO, Sandanger TM (2014). Repeated measurements of per- and polyfluoroalkyl substances (PFASs) from 1979 to 2007 in males from Northern Norway: assessing time trends, compound correlations and relations to age/birth cohort. Environ Int.

[CR40] NTP (National Toxicology Program) (2019a) NTP technical report on the toxicity studies of perfluoroalkyl carboxylates (perfluorohexanoic acid, perfluorooctanoic acid, perfluorononanoic acid, and perfluorodecanoic acid) administered by gavage to Sprague Dawley (Hsd:Sprague Dawley SD) rats. NTP TOX 97. Research Triangle Park, North Carolina, USA. https://ntp.niehs.nih.gov/sites/default/files/ntp/htdocs/st_rpts/tox097_508.pdf. Accessed 18 Dec 2023

[CR41] NTP (National Toxicology Program) (2019b) NTP technical report on the toxicity studies of perfluoroalkyl sulfonates (perfluorobutane sulfonic acid, perfluorohexane sulfonate potassium salt, and perfluorooctane sulfonic acid) administered by gavage to Sprague Dawley (Hsd:Sprague Dawley SD) rats. NTP TOX 96. Research Triangle Park, North Carolina, USA. https://ntp.niehs.nih.gov/sites/default/files/ntp/htdocs/st_rpts/tox096_508.pdf. Accessed 18 Dec 2023

[CR42] Ojo AF, Peng C, Ng JC (2021). Assessing the human health risks of per- and polyfluoroalkyl substances: a need for greater focus on their interactions as mixtures. J Hazard Mater.

[CR43] Pant A, Rondini EA, Kocarek TA (2019). Farnesol induces fatty acid oxidation and decreases triglyceride accumulation in steatotic HepaRG cells. Toxicol Appl Pharmacol.

[CR44] R Core Team (2020) R: a language and environment for statistical computing. R Foundation for Statistical Computing, Vienna

[CR45] Rogue A, Lambert C, Josse R (2011). Comparative gene expression profiles induced by PPARgamma and PPARalpha/gamma agonists in human hepatocytes. PLoS One.

[CR46] Rosen MB, Das KP, Rooney J, Abbott B, Lau C, Corton JC (2017). PPARα-independent transcriptional targets of perfluoroalkyl acids revealed by transcript profiling. Toxicology.

[CR47] Rylander C, Dumeaux V, Olsen KS, Waaseth M, Sandanger TM, Lund E (2011). Using blood gene signatures for assessing effects of exposure to perfluoroalkyl acids (PFAAs) in humans: the NOWAC postgenome study. Int J Mol Epidemiol Genet.

[CR48] Scharmach E, Buhrke T, Lichtenstein D, Lampen A (2012). Perfluorooctanoic acid affects the activity of the hepatocyte nuclear factor 4 alpha (HNF4alpha). Toxicol Lett.

[CR49] Schlezinger JJ, Hyötyläinen T, Sinioja T, Boston C, Puckett H, Oliver J, Heiger-Bernays W, Webster TF (2021). Perfluorooctanoic acid induces liver and serum dyslipidemia in humanized PPARα mice fed an American diet. Toxicol Appl Pharmacol.

[CR50] Sen P, Qadri S, Luukkonen PK (2022). Exposure to environmental contaminants is associated with altered hepatic lipid metabolism in non-alcoholic fatty liver disease. J Hepatol.

[CR51] Soderstrom S, Lille-Langoy R, Yadetie F (2022). Agonistic and potentiating effects of perfluoroalkyl substances (PFAS) on the Atlantic cod (*Gadus morhua*) peroxisome proliferator-activated receptors (Ppars). Environ Int.

[CR52] Vinken M, Landesmann B, Goumenou M, Vinken S, Shah I, Jaeschke H, Willett C, Whelan M, Rogiers V (2013). Development of an adverse outcome pathway from drug-mediated bile salt export pump inhibition to cholestatic liver injury. Toxicol Sci.

[CR53] Wang Z, DeWitt JC, Higgins CP, Cousins IT (2017). A never-ending story of per- and polyfluoroalkyl substances (PFASs)?. Environ Sci Technol.

[CR54] Weber F, Freudinger R, Schwerdt G, Gekle M (2005). A rapid screening method to test apoptotic synergisms of ochratoxin A with other nephrotoxic substances. Toxicol In Vitro.

[CR55] Wolf CJ, Rider CV, Lau C, Abbott BD (2014). Evaluating the additivity of perfluoroalkyl acids in binary combinations on peroxisome proliferator-activated receptor-alpha activation. Toxicology.

