# Supplementary material for: Impact of perfluoroalkyl substances (PFAS) and PFAS mixtures on lipid metabolism in differentiated HepaRG cells as a model for human hepatocytes
Source: Arch Toxicol. 2023 Dec 20;98(2):507–24. doi: 10.1007/s00204-023-03649-3 (PMC10794458; doi:10.1007/s00204-023-03649-3)
Supplement: Supplementary file 2 — Supplementary file2 (DOCX 131 kb) [file 204_2023_3649_MOESM2_ESM.docx]

**b**

**a**

**d**

**c**

**f**

**e**

Supplementary Fig. 2: Activation of PXR and CAR nuclear receptors in HEK293T cells after 24 h incubation with different concentrations of PFOS, PFHxS, and the Ronneby mixture. PXR activation is shown in a, c and e (a: PFOS, c: PFHxS, and e: Ronneby), and CAR activation is shown in b, d and f (b: PFOS, d: PFHxS, and f: Ronneby). Nuclear receptor activation was expressed as the ratio of firefly luciferase signals normalized to *Renilla* luciferase signals. The positive control (PC) for PXR was 10 μM SR12813, and the PC for CAR was 10 μM CITCO. The solvent control (SC) consisted of medium with 1% DMSO. Statistical analysis was conducted using one-way ANOVA followed by Dunnett’s test (* p < 0.05, ** p < 0.01 and *** p < 0.001).
