# Supplementary material for: Impact of perfluoroalkyl substances (PFAS) and PFAS mixtures on lipid metabolism in differentiated HepaRG cells as a model for human hepatocytes
Source: Arch Toxicol. 2023 Dec 20;98(2):507–24. doi: 10.1007/s00204-023-03649-3 (PMC10794458; doi:10.1007/s00204-023-03649-3)
Supplement: Supplementary file 3 — Supplementary file3 (DOCX 21 kb) [file 204_2023_3649_MOESM3_ESM.docx]

**Supplementary Table 1: List of primers**

|  |  |  |
| --- | --- | --- |
|  | Primer sequence (5′–3′) |  |
|  | Forward | Reverse |
|  |  |  |
| ACA2 | ACTTGGCTTACTTGAGAACAGGA | CAGTCAGTGGCATCTCACCTA |
| ACTB | CCTTGCACATGCCGGAG | GCACAGAGCCTCGCCTT |
| ADH4 | GGGTTGTAAAGCAGCAGGAG | AGGATTGAGGCAGTCAGTGG |
| ANXA10 | TGGAGTGCTCCTCCTAGCAT | CGATCAAATATTTTCATCCCTGA |
| ARG1 | GTTTCTCAAGCAGACCAGCC | GCTCAAGTGCAGCAAAGAGA |
| CCL20 | CGTGTGAAGCCCACAATAAA | CAAGAGTTTGCTCCTGGCTG |
| CD36 | CAAATCAACAGCAAGACATGAA | GCAAGACTCTGGAGCCAGTC |
| CPT1A | ACGCTACTTCAAGGTCTGGC | GGCTCCGAGGTATTGTCCAG |
| CYP3A4 | TCACAAACCGGAGGCCTTTT | TGGTGAAGGTTGGAGACAGC |
| CYP7A1 | GACACACCTCGTGGTCCTCT | TTTCATTGCTTCTGGGTTCC |
| CYP2B6 | TTCCTACTGCTTCCGTCTATCAAA | GTGCAGAATCCCACAGCTCA |
| CYP2D6 | GCCTTCCTGCCTTTCTCAGCAG | ATGGGCTCACCAGGAAAGCAAA |
| FABP1 | CAAGTTCACCATCACCGCTGGGTC | TCATTGTCTCCAGCTCACATTCCTC |
| FASN | ACAGCGGGGAATGGGTACT | GACTGGTACAACGAGCGGAT |
| GAPDH | TTAAAAGCAGCCCTGGTGAC | CTCTGCTCCTCCTGTTCGAC |
| HMGCR | GACGTGAACCTATGCTGGTCAG | GGTATCTGTTTCAGCCACTAAGG |
| HMGCS2 | GTAGCCCCATAAGCATCAGC | TAGCACCATAAGCCCAGGAC |
| INSIG1 | TCCTTGCTCTCAGAATCGGT | CGTTCTTGGCTCCCTTGTAT |
| PDK4 | AGGTGGTGTTCCCCTGAGA | AACCAAAACCAGCCAAAGGAG |
| PLIN2 | ACTGGCTGGTAGGTCCCTTT | GTCTCCTGGCTGCTCTTGTC |
| POR | GATGTTCCTCCCCGTTTTCT | TCATCGTGGGTCTCCTAACC |
| SLCO1B1 | GCCAAGAACATCTTCAATCCA | TCAAACTGAGCATCAACAACAA |
| SREBF1 | CGGAACCATCTTGGCAACAGT | CGCTTCTCAATGGCGTTGT |
| 18SrRNA | TTCCAATTACAGGGCCTCGA | CCTGAGAAACGGCTACCACT |

**Supplementary Information on gene selection**

For gene expression analysis, genes were selected according to published data on gene expression in HepaRG cells. A set of ten marker genes for steatosis was taken from Lichtenstein et al. (2020b). A set of 13 genes associated with lipid and cholesterol metabolism in HepaRG cells including several PPARα and PPARγ target genes was selected according to the publications from Louisse et al. (2020), Pant et al. (2019) and Rogue et al. (2011).

Steatosis marker genes

ANXA10 – annexin A10

ANXA10 is a member of the annexin family encoding a calcium-dependent phospholipid-binding protein. Annexins play a role in the regulation of cellular growth and in different signal transduction pathways. The exact function of ANXA10 is not determined yet.

ARG1 – arginase 1

ARG1 catalyzes the hydrolysis of arginine to ornithine and urea. The enzyme encoded by ARG1 is a cytosolic protein that is predominantly expressed in the liver as a component of the urea cycle.

CCL20 – C-C motif chemokine ligand 20

CCL20 belongs to the subfamily of small cytokine CC genes that encode for proteins involved in immunoregulatory and inflammatory processes. The protein encoded by CCL20 displays chemotactic activity for lymphocytes and can repress proliferation of myeloid progenitors. It is predominantly expressed in gall bladder, but also in liver and intestine.

CD36 – CD36 molecule

The protein encoded by CD36 is a major glycoprotein of the platelet surface and serves as a receptor for thrombospondin. Moreover, it has various functions as adhesion molecule in different tissues as it also binds to collagen, anionic phospholipids, oxidized LDL and long-chain fatty acids. CD36 is regulated by PPARα and is involved in the regulation of fatty acid transport.

CYP2D6 – cytochrome P450 family 2 subfamily D member 6

The protein encoded by CYP2D6 is a member of the cytochrome P450 superfamily. It is a monooxygenase being involved in many oxidative reactions in drug metabolism and in synthesis of cholesterol, steroids and other lipids. CYP2D6 is mainly expressed in liver.

FASN – fatty acid synthase

The protein encoded by FASN is an enzyme catalyzing the synthesis of palmitate from acetyl-CoA and malonyl-CoA, in the presence of NADPH, into long-chain saturated fatty acids. FASN is mainly expressed in fat tissue.

INSIG1 – insulin induced gene 1

The protein encoded by INSIG1 is an endoplasmatic reticulum membrane protein that is involved in the regulation of cholesterol metabolism, lipogenesis, and glucose homeostasis. It is essential for the sterol-mediated trafficking of several proteins via the endoplasmic reticulum. INSIG1 is mainly expressed in liver.

POR – cytochrome P450 oxidoreductase

The protein encoded by POR is an endoplasmic reticulum membrane oxidoreductase that is essential for multiple metabolic processes, including reactions catalyzed by cytochrome P450 proteins for metabolism of steroid hormones, drugs and xenobiotics. POR is ubiquitously expressed in various tissues.

SLCO1B1 – solute carrier organic anion transporter family member 1B1

The protein encoded by SLCO1B1 is a liver-specific member of the organic anion transporter family. It is a transmembrane receptor that mediates the sodium-independent uptake of numerous endogenous compounds, e. g. bilirubin, and numerous xenobiotics from the blood into the hepatocyte.

SREBF1 – sterol regulatory element binding transcription factor 1

SREBF1 encodes a transcription factor that binds to the sterol regulatory element-1 (SRE1), which is a motif that is found in the promoters of several genes involved in sterol biosynthesis. SREBF1 is ubiquitously expressed in various tissues.

PPARα target genes

ACAT2 – acetyl-CoA acetyltransferase 2

The protein encoded by ACAT2 is a cytosolic acetoacetyl-CoA thiolase that is involved in lipid metabolism. ACAT2 is ubiquitously expressed in various tissues.

ADH4 – alcohol dehydrogenase 4 (class II), pi polypeptide

The protein encoded by ADH4 is a member of the alcohol dehydrogenase family that facilitates oxidation of a wide variety of substrates, including long-chain aliphatic alcohols and aromatic alcohols. ADH4 is expressed in liver.

CPT1A – carnitine palmitoyltransferase 1A

The protein encoded by CPT1A is involved in mitochondrial oxidation of long-chain fatty acids. It is located in the outer mitochondrial membrane. CPT1A is ubiquitously expressed in various tissues.

PDK4 – pyruvate dehydrogenase kinase 4

The protein encoded by PDK4 is a member of the PDK/BCKDK protein kinase family that is located in the matrix of mitochondria. Its function is to inhibit the pyruvate dehydrogenase complex by phosphorylating one of its subunits, thereby contributing to the regulation of glucose metabolism. PDK4 is expressed in various tissues.

PLIN2 – perilipin 2

The protein encoded by PLIN2 belongs to the perilipin family and is involved in the coating of intracellular lipid storage droplets. It is not restricted to adipocytes, but is found in many tissues, suggesting that it may serve as a marker of lipid accumulation in diverse cell types and diseases.

Genes associated with cholesterol metabolism

CYP7A1 – cytochrome P450 family 7 subfamily A member 1

The protein encoded by CYP7A1 is an endoplasmic reticulum membrane protein that catalyzes the first reaction in the cholesterol catabolic pathway in the liver, which converts cholesterol to bile acids. This reaction is the rate-limiting step and the major site of regulation of bile acid synthesis. CYP7A1 expression is restricted to liver.

HMGCR – 3-hydroxy-3-methylglutaryl-CoA reductase

The protein encoded by HMGCR catalyzes the rate-limiting step in cholesterol synthesis. It is inhibited by its own product, mevalonate, thereby regulating cholesterol synthesis. HMGCR is ubiquitously expressed in various tissues.

HMGCS2 – 3-hydroxy-3-methylglutaryl-CoA synthase 2

The protein encoded by HMGCS2 is a mitochondrial enzyme that catalyzes the first reaction of ketogenesis, a metabolic pathway that provides lipid-derived energy during times of carbohydrate deprivation. HMGCS2 is mainly expressed in liver.

Genes associated with lipid and xenobiotic metabolism

CYP2B6 – cytochrome P450 family 2 subfamily B member 6

The protein encoded by CYP2B6 is a member of the cytochrome P450 superfamily of enzymes. It is located in the endoplasmic reticulum and it is known to oxidize some xenobiotics. Expression of CYP2B6 is restricted to liver.

CYP3A4 – cytochrome P450 family 3 subfamily A member 4

The protein encoded by CYP3A4 belongs to the family of cytochrome P450 monooxygenases. It localizes in the endoplasmic reticulum and is involved in the metabolism of a large variety of drugs, xenobiotics, and steroids. CYP3A4 is expressed in liver and intestine.

FABP1 – fatty acid binding protein 1

The protein encoded by FABP1 is a cytosolic protein that binds long-chain fatty acids, bile acids, and other hydrophobic ligands. It has a function in fatty acid uptake, transport, and metabolism. FABP1 is expressed in liver and intestine.

UGT1A1 – UDP glucuronosyltransferase family 1 member A1

The protein encoded by UGT1A1 is an enzyme of the glucuronidation pathway that transforms small lipophilic molecules, e. g. bilirubin and C18 steroids, into water-soluble, excretable metabolites. UGT1A1 is expressed in liver, kidney, and intestine.

Housekeeping genes

ACTB – actin beta

GAPDH – glyceraldehyde-3-phosphate dehydrogenase

18SrRNA – 18S ribosomal RNA

In qRT-PCR studies, ACTB, GAPDH and 18SrRNA are commonly used as housekeeping genes for normalization purposes.
